# Piperlongumine Inhibits Migration of Glioblastoma Cells via Activation of ROS-Dependent p38 and JNK Signaling Pathways

**DOI:** 10.1155/2014/653732

**Published:** 2014-05-22

**Authors:** Qian Rong Liu, Ju Mei Liu, Yong Chen, Xiao Qiang Xie, Xin Xin Xiong, Xin Yao Qiu, Feng Pan, Di Liu, Shang Bin Yu, Xiao Qian Chen

**Affiliations:** ^1^Department of Pathophysiology, School of Basic Medicine, Key Laboratory of Neurological Diseases, Ministry of Education and Hubei Provincial Key Laboratory of Neurological Diseases, Tongji Medical College, Huazhong University of Science and Technology, Wuhan 430030, China; ^2^Department of Urology, Union Hospital, Huazhong University of Science and Technology, Wuhan 430022, China

## Abstract

Piperlongumine (PL) is recently found to kill cancer cells selectively and effectively via targeting reactive oxygen species (ROS) responses. To further explore the therapeutic effects of PL in cancers, we investigated the role and mechanisms of PL in cancer cell migration. PL effectively inhibited the migration of human glioma (LN229 or U87 MG) cells but not normal astrocytes in the scratch-wound culture model. PL did not alter EdU^+^-cells and cdc2, cdc25c, or cyclin D1 expression in our model. PL increased ROS (measured by DCFH-DA), reduced glutathione, activated p38 and JNK, increased I**κ**B**α**, and suppressed NF**κ**B in LN229 cells after scratching. All the biological effects of PL in scratched LN229 cells were completely abolished by the antioxidant N-acetyl-L-cysteine (NAC). Pharmacological administration of specific p38 (SB203580) or JNK (SP600125) inhibitors significantly reduced the inhibitory effects of PL on LN229 cell migration and NF**κ**B activity in scratch-wound and/or transwell models. PL prevented the deformation of migrated LN229 cells while NAC, SB203580, or SP600125 reversed PL-induced morphological changes of migrated cells. These results suggest potential therapeutic effects of PL in the treatment and prevention of highly malignant tumors such as glioblastoma multiforme (GBM) in the brain by suppressing tumor invasion and metastasis.

## 1. Introduction

Reactive oxygen species (ROS) plays a crucial role in the processes of tumor genesis, progression, and metastasis. Cancer cells usually possess higher levels of ROS and higher antioxidant activities in an uncontrolled status as compared to normal cells [1]. As a result, cancer cells are unable to cope with additional oxidative stress and become vulnerable to ROS [2, 3]. Therefore, targeting ROS is an important therapeutic strategy for cancer as exemplified by cancer drugs such as daunorubicin [4], vincristine [5], paclitaxel, docetaxel, and 2-methoxyestradiol [6]. Exploring the mechanisms of ROS-based treatment is required for further improving the efficacy and specificity of cancer drugs [3].

Piperlongumine (piplartine, PL), a primary constituent of* Piper longum* (long pepper), exists in the fruits and roots of the plant [7]. It is reported that PL possesses analgesic [8], antidepressant [9], cardioprotective [10], antiplatelet [11, 12], anti-inflammatory [13], and antiatherosclerotic [14] effects. Recently, PL is identified as a highly selective cytotoxic compound for cancer cells but not normal cells [15]. PL directly binds to and inhibits the antioxidant enzyme glutathione S-transferase pi 1 (GSTP1), resulting in elevated levels of ROS and subsequent cancer-selective cell death [15]. Accumulating evidence has suggested that PL might be a potential chemotherapeutic in human cancer [15–18].

Metastasis is the main cause of mortality in cancer while the key process of metastasis is the migration of cancer cells to invade adjacent tissues [19, 20]. Glioblastoma multiforme (GBM), a grade IV astrocytoma, is the most common and most aggressive malignant primary brain tumor in humans with a median survival time of less than 14 months after routine radiation therapy and chemotherapy following resection [21]. Due to the confined space of the brain and highly integrity of neuronal-astrocytic interactions, the invasion of glioma cells is particularly devastating. We have recently reported that PL can selectively kill GBM cells but not normal astrocytes [22]. To further explore the therapeutic or preventive effects of PL in GBM, we investigated the biological effects and mechanisms of PL in GBM cell migration.

In this study, we demonstrated that PL inhibited GBM cell migration effectively. The mechanisms of PL's action included ROS accumulation, p38 and JNK activation, and NF*κ*B nuclear translocation.

## 2. Materials and Methods

### 2.1. Reagents

Piperlongumine (PL), N-acetyl-L-cysteine (NAC), 2,7-dichlorodihydrofluorescein diacetate (DCFH-DA), and Hoechst 33342 were purchased from Sigma-Aldrich (St. Louis, MO, USA). SB203580 and SP600125 were purchased from Cell Signaling Technology, Inc. (Danvers, MA, USA). DMEM and fetal bovine serum (FBS) were purchased from Life Technologies, Inc. (Grand Island, NY, USA). Antibodies against NF*κ*B p65, I*κ*B*α*, glyceraldehyde-3-phosphate dehydrogenase (GAPDH), and *β*-actin were purchased from Santa Cruz Biotechnology (Heidelberg, Germany), and the antibodies against p-p38, total p38, p-JNK, and total-JNK were from Cell Signaling Technology.

### 2.2. Cell Culture and Drug Treatments

Human GBM cell lines LN229 and U87 MG (U87) were kindly provided by Prof. Haian Fu (Emory University, GA, USA) and were cultured in DMEM containing 5% or 10% FBS, respectively. Primary cultures of rat cerebral cortical astrocytes were cultured as described previously [23]. All cells were maintained in a humidified atmosphere of 95% air and 5% CO_2_ at 37°C.

PL was dissolved in dimethyl sulfoxide (DMSO) at a stock concentration of 50 mM. The final concentration of PL was 0–20 *μ*M with a maximal DMSO concentration less than 0.05%. DMSO of the same concentration in PL solutions was used as the vehicle control. The antioxidant NAC (3 mM), SB203580 (p38 pathway specific inhibitor, 10 *μ*M), and SP600125 (JNK pathway specific inhibitor, 10 *μ*M) were added 2 h before PL treatment.

### 2.3. Scratch-Wound Culture Model and Cell Migration Assay

Cancer cells were planted in a 35 mm dish and incubated for 24 h to form a monolayer with confluence. For wound-healing study, monolayer cells were scratched with a 200 *μ*L pipette tip and the wound edges were micrographed and were treated with PL immediately; then the wound edges were remicrographed 24 h after PL treatment. To investigate the earlier cell migration (3 or 6 h) after scratching, GBM cells were pretreated with PL for 6 h and then monolayer cells were scratched. After the wounded edges were micrographed immediately after scratching, the cultures were incubated for another 3 or 6 h in DMEM containing 20% FBS [24]. Cells were then fixed with 4% paraformaldehyde for 30 min, stained by Hoechst 33342, and micrographed. The width of wound (i.e., the gap between the two opposite wound edges) was measured using the Image-Pro Plus software. The relative migration rate was calculated by the reduction of the wound width between immediately after scratch and 6 h after scratch. This value reflects the distance migrated by the leading edge of the wound during the given time interval.

### 2.4. Measurement of ROS

Intracellular ROS production was determined by 2,7-dichlorodihydrofluorescein diacetate (DCFH-DA) staining followed by fluorescent microscope [22]. Briefly, cells were incubated with 10 *μ*M DCFH-DA solution at 37°C for 0.5 h, washed with PBS twice, and photographed under a conventional fluorescent microscope (Olympus, Tokyo, Japan). For each culture, a minimum of 9 random fields were captured.

### 2.5. Measurement of Reduced Glutathione

Treated cells were washed twice with PBS, scraped off the plates, and then subjected to sonication. Protein concentration was assessed by using bicinchoninic acid assay kit (Beyotime, Nantong, China) and reduced glutathione (GSH) was measured by using a kit (Nanjing Jiangcheng Bioengineering Institute, Nanjing, China) according to the manufacturer's instructions. Results are expressed as *μ*mol of GSH per gram of protein (*μ*mol/g prot) [25].

### 2.6. Western Blot Analysis

Western blot analysis was performed as described previously [26, 27]. Briefly, cells were seeded in 35 mm dishes and incubated for 24 h. After PL treatment for 6 h, cells were scratched and incubated in DMEM supplemented with 20% FBS for another 3 h. For the whole cell lysates, the cells were gathered in RIPA lysis buffer (Beyotime, Nantong, China) and sonicated. The supernatants were removed as protein samples after centrifugation. Cytosolic and nuclear extract were isolated by using a kit from Sangon Biotech (Shanghai, China) according to the manufacturer's instructions. Equal amounts of total soluble proteins were subjected to western blot analysis. The blots were visualized with corresponding fluorescent secondary antibodies and the bands were quantified by using the Odyssey Infrared Imaging System (LI-COR Bioscience, USA).

### 2.7. Transwell Cell Migration Assay

LN229 cells were seeded at the concentration of 1 × 10^5^ per well into the upper chamber of a transwell apparatus with an 8 *μ*m pore size membrane (BD Bioscience, NJ, USA) in DMEM containing drugs of determined concentration. The medium of lower chamber was changed to DMEM containing 10% FBS 6 h after the initial cell seeding. After another 24 h incubation, cells remaining in the upper surface of the filter were scraped off with cotton swabs. Cells migrating to the lower surface of the filter were stained with Giemsa, examined by microscopy, and photographed. The migrated cell number was expressed as the average number of migrated cells per microscopic field (×50) over seven fields.

### 2.8. EdU (5′-Ethynyl-2′-deoxyuridine) Labeling

LN229 cells (2 × 10^5^) were plated in each well of 12-well plates. EdU (10 *μ*M) from the EdU Kit (Ribobio, Guangzhou, China) was used for labeling according to the manufacturer's instructions as reported previously [27].

### 2.9. Statistical Analyses

All experiments were repeated independently at least three times. The values were expressed as mean ± SEM and statistics were performed with a 2-way ANOVA followed by the Student-Newman-Keuls test. *P* values of less than 0.05 were considered statistically significant.

## 3. Results

### 3.1. PL Inhibits Migration of GBM Cells but Not Normal Astrocytes in Cultures after Scratch

**Figure 1:**
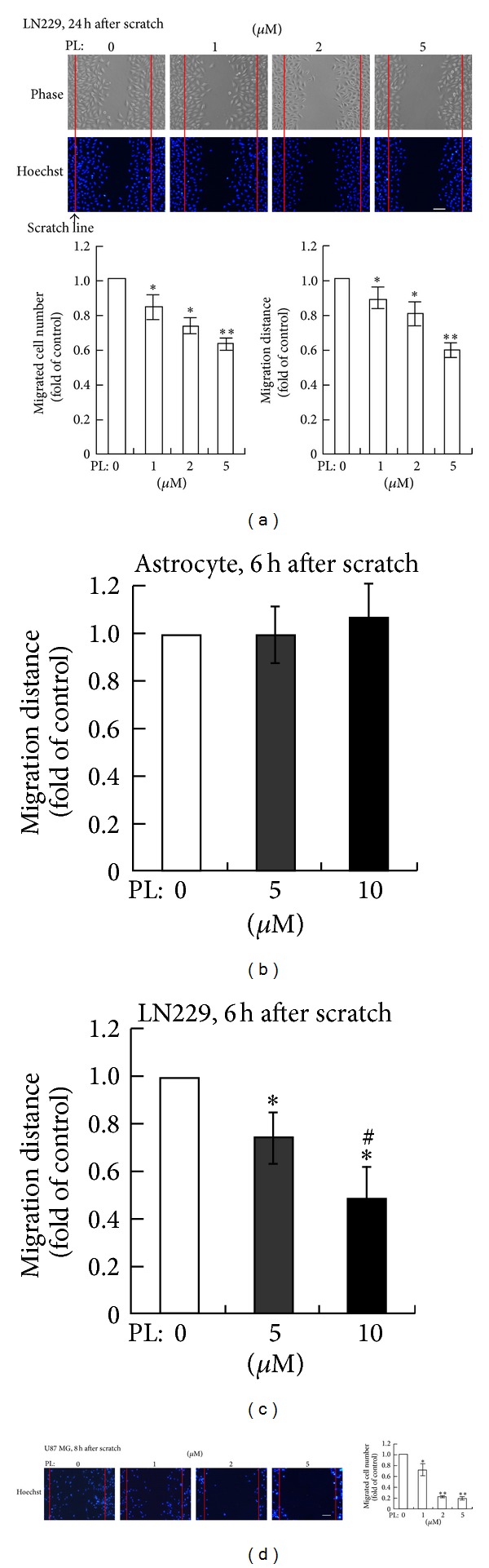
PL inhibits migration of GBM cells but not normal astrocytes in scratch-wound assay in vitro. (a) Effects of PL on wound-healing 24 h after scratch in LN229 cells. LN229 cells in confluence were scratched, washed twice with DMEM, and then treated with different concentration (0, 1, 2, or 5 *μ*M) of PL for 24 h. The cultures were fixed and stained with Hoechst 33342. The red lines indicated the scratch lines. The number of migrated cells between the opposite scratch lines was counted and the distance migrated by the cells at the leading edge was measured. *P < 0.05 and **P < 0.01 compared to group of 0 *μ*M of PL. (b) Effects of PL on cell migration in primary cultured astrocytes. Cultured astrocytes in confluence were treated with different concentration (0, 5, or 10 *μ*M) of PL for 6 h before cell scratch. The migration distance was measured 6 h after scratching. (c) Effects of PL on cell migration in LN229 cells. Migration of LN229 cells was measured 6 h after scratching upon PL treatment. *P < 0.05 compared to group of 0 *μ*M of PL; ^#^ *P* < 0.05 compared to group of 5 *μ*M of PL. (d) Effects of PL on cell migration in U87 MG cells at 8 h after scratching upon PL pretreatment. *P < 0.05 and **P < 0.01 compared to group of 0 *μ*M of PL. Bar: 50 *μ*m.

We tested the effects of PL on cell migration in GBM cell lines by using the well-known scratch-wound model. After confluent LN229 cells were scratched (the scratch line was indicated by the red line in Figure 1(a), upper panels), PL was administrated immediately. Clearly, the cell numbers and the migration distances were decreased between the opposite scratch lines at 24 h after scratching at various concentrations (1, 2, or 5 *μ*M) of PL (Figure 1(a)). To reduce the effect of cell proliferation after scratching, cell migration was measured at 6 h after scratching with PL pretreatment (6 h before scratching). In normal cultured astrocytes (3-4 weeks in cultures), PL at 5 and 10 *μ*M did not affect the migration of astrocytes (Figure 1(b)). In LN229 cells, PL significantly reduced the migration distance of LN229 cells in a dose-dependent manner (0, 5, and 10 *μ*M) (Figure 1(c)). Since PL at 5 and 10 *μ*M does not cause cell death of GBM cells or astrocytes within 6 h [22], these data revealed a selective inhibitory effect of PL on the migration of GBM cells.

In addition to LN229 cells, we examined the effect of PL on cell migration in another GBM cell line, which are U87 MG cells. Results of Hoechst staining clearly showed that migrated cells were reduced evidently at 8 h after scratching upon various concentrations (1, 2, and 5 *μ*M) of PL (Figure 1(d), upper panels). Statistical analysis demonstrated that PL significantly reduced the numbers of migrated U87 cells in a dosage-dependent manner (Figure 1(d), lower panel).

### 3.2. Effects of PL on LN229 Cell Proliferation

**Figure 2:**
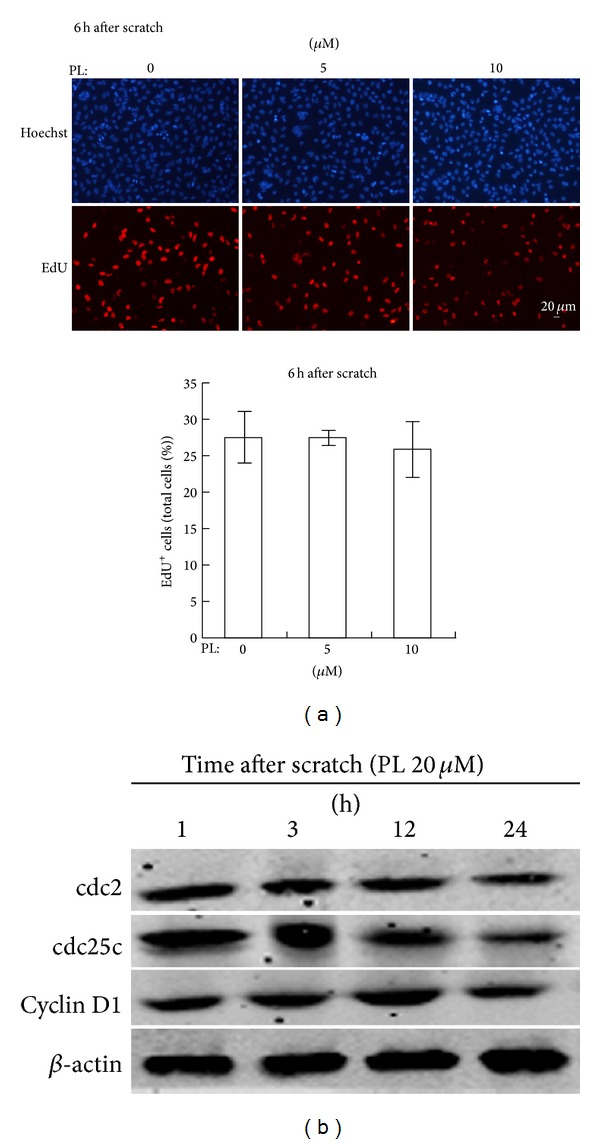
Effects of PL on cell proliferation in LN229 cells. (a) Effects of PL on EdU^+^-stained cells at 6 h after scratching upon PL treatment. (b) Representative western blot results of cdc2, cdc25c, and cyclin D1 at various time points (1, 3, 12, and 24 h) after scratching upon 20 *μ*M of PL treatment in LN229 cells. *β*-actin was used as a loading control.

To exclude the possible effects of cell proliferation after scratching and PL treatment in our assay, we measured newly divided LN229 cells at 6 h after scratching with PL pretreatment. Results of EdU staining demonstrated that the percentage of EdU^+^-stained cells (representing newly divided cells) was not altered at 6 h after scratching upon 5 or 10 *μ*M of PL (Figure 2(a)). Further, results of western blot showed that expression of cell cycle-associated proteins such as cdc2, cdc25C, and cyclin D1 was not altered evidently at 1, 3, or 12 h after scratching upon 20 *μ*M PL (Figure 2(b)). These evidences suggested that cell migration but not cell cycle or cell proliferation was the major factor contributing to the reduced migrated cells in the scratched area upon PL treatment within the time-scale in our model.

### 3.3. PL Inhibits GBM Cell Migration via ROS Accumulation

**Figure 3:**
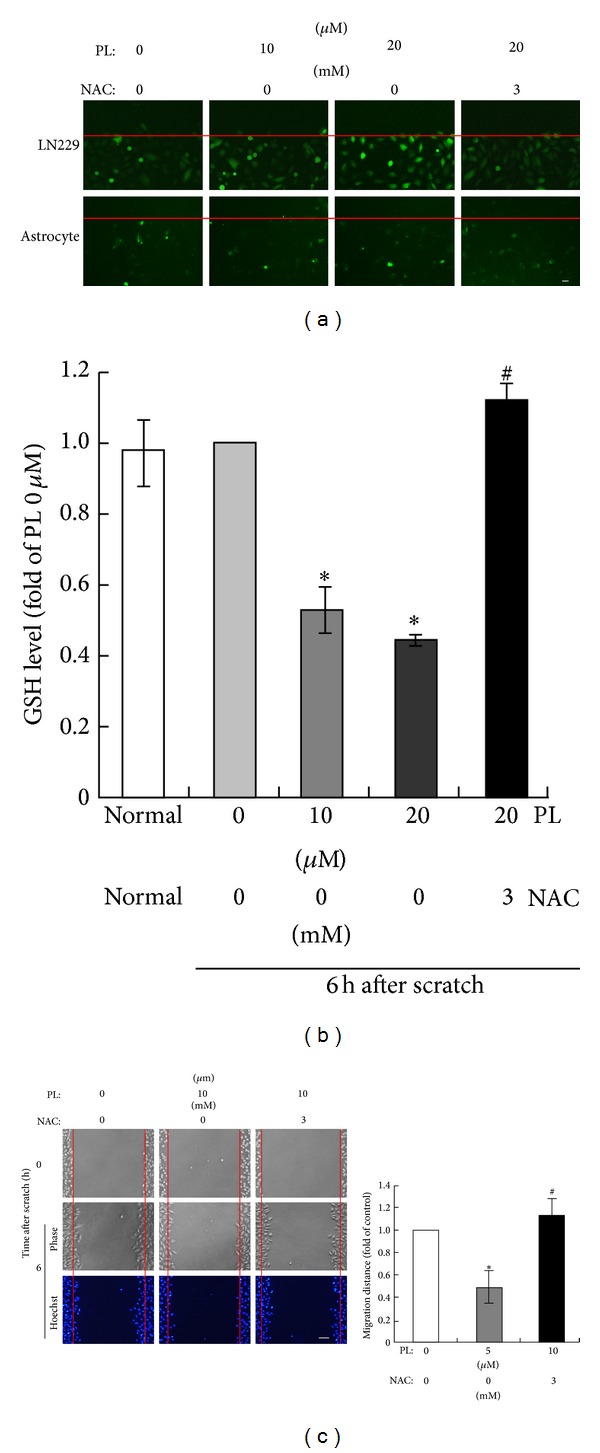
PL suppresses migration of LN229 cells via ROS accumulation. (a) PL increased ROS levels in LN229 cells but not in astrocytes after scratch. LN229 cells or cultured astrocytes were scratched and then treated with 0, 10, or 20 *μ*M PL for 3 h and then stained with DCFH-DA. NAC (3 mM) was administrated 2 h before cell scratch. Representative micrographs showed that the fluorescent intensity of DCFH-DA was enhanced after PL treatment. The red lines indicated the initial edges of scratches. Bar: 20 *μ*m. (b) PL reduced GSH in LN229 cells after scratch. LN229 cells were scratched and then treated with 0, 10, or 20 *μ*M PL for 3 h and the cellular GSH level was measured. Normal, unscratched cultures without PL treatment. *P < 0.05 compared to group of 0 *μ*M of PL; ^#^ *P* < 0.05 compared to group of 20 *μ*M of PL. (c) Effects of PL and NAC on LN229 cell migration after cell scratch. The red lines indicated the initial edges of scratches. Bar: 50 *μ*m. *P < 0.05 compared to group of DMSO; ^#^ *P* < 0.05 compared to group of 10 *μ*M of PL.

Since PL exhibited a selective inhibitory effect on GBM cell migration, we further investigated its underlying mechanisms. Previous studies have suggested that PL exerts its anticancer effects via increasing ROS [15, 22, 28, 29]; we speculated that PL might suppress GBM cell migration via inducing ROS. LN229 cells in confluence were treated with PL immediately after cell scratch and intracellular ROS was detected by specific ROS marker DCFH-DA. The fluorescent intensities of DCFH-DA (representing intracellular ROS levels) were evidently enhanced in LN229 cells along the scratch line (indicated by the red line) 3 h after PL treatment (10 and 20 *μ*M) (Figure 3(a)). Pretreatment of antioxidant NAC (3 mM) reduced DCFH-DA fluorescence to the control level (PL 0 *μ*M) (Figure 3(a)). Consistent to the increase of ROS, treatment with PL for 3 h significantly reduced cellular GSH levels in scratched LN229 cells in a dosage-dependent manner (Figure 3(b)). The reduction of GSH by PL was completely reversed in the presence of NAC (Figure 3(b)). These data demonstrated that PL induced ROS accumulation in scratched LN229 cells.

We then tested whether ROS accumulation contributed to the PL-inhibited cell migration in LN229 cells. Morphological micrographs (phase) and nuclear staining (Hoechst) clearly showed that LN229 cells were migrating toward the nude areas of wound from the scratch edges (indicated by red lines) and cell numbers were increased in the wound areas 6 h after scratch (Figure 3(c)). PL at 10 *μ*M evidently reduced cell migration and the migrated cells (Figure 3(c)). NAC completely reversed the inhibitory effects of PL on LN229 cell migration (Figure 3(c)). Statistical analysis demonstrated that the migration distance of LN229 cells was significantly reduced by PL (10 *μ*M) but this effect was completely abolished by NAC (Figure 3(c)). These data demonstrated that PL suppressed LN229 cell migration via ROS accumulation.

### 3.4. PL Suppresses LN229 Cell Migration via ROS-Dependent p38 and JNK Activation

**Figure 4:**
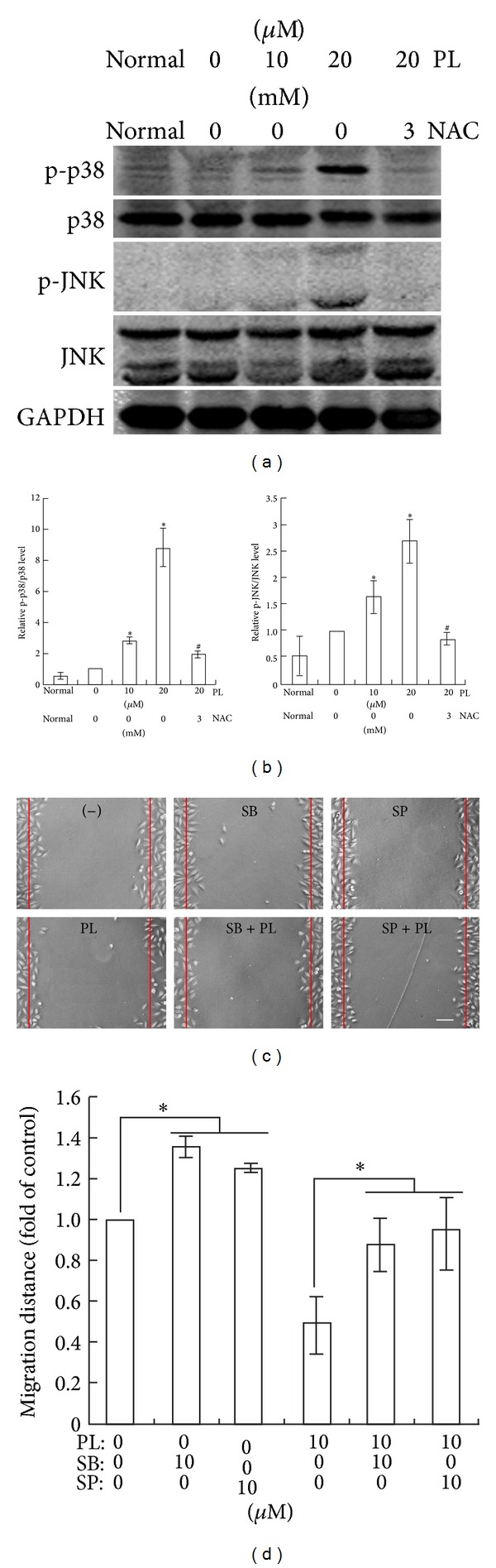
Activation of p38 and JNK by ROS accumulation contributes to PL's effect on LN229 cell migration. (a) Representative western blots of JNK and p38 phosphorylation at 3 h after scratch in PL-treated LN229 cells. GAPDH was used as a loading control. (b) Statistical analysis of p38 and JNK phosphorylation of western blot results. Normal, unscratched cultures without PL treatment. *P < 0.05 compared to group of 0 *μ*M of PL; ^#^ *P* < 0.05 compared to group of 20 *μ*M of PL treatment alone. (c) Representative micrographs showing the effects of PL on LN229 cell migration at 6 h after scratch in the presence of PL, SB203580, or SP600125. SB203580 (10 *μ*M) and SP600125 (10 *μ*M) were administrated 2 h before PL treatment. Bar: 50 *μ*m. (d) Statistical analysis of migrated distance of LN229 cells upon PL, SB203580, or SP600125. *P < 0.05.

To address how ROS accumulation causes PL-suppressed LN229 cell migration, we analyzed the effects of PL on p38 and JNK activation, two classical ROS-activated signaling pathways, in scratched LN229 cells. Western blot demonstrated that PL significantly increased the levels of p-p38 and p-JNK in a dosage-dependent manner in LN229 cells 3 h after scratch while the scratch injury itself did not alter p-p38 and p-JNK levels (Figures 4(a) and 4(b)). Pretreatment of NAC completely abolished the PL-induced p38 and JNK activation (Figures 4(a) and 4(b)). We then tested whether the activation of p38 and JNK contributed to the effects of PL on LN229 cell migration. Pretreatment of SB203580 and SP600125 increased LN229 cell migration 6 h after cell scratch (Figure 4(c)). Statistical analysis demonstrated that the inhibition of both p38 and JNK pathways significantly increased the migration distance of LN229 cells upon PL treatment (Figure 4(d)).

**Figure 5:**
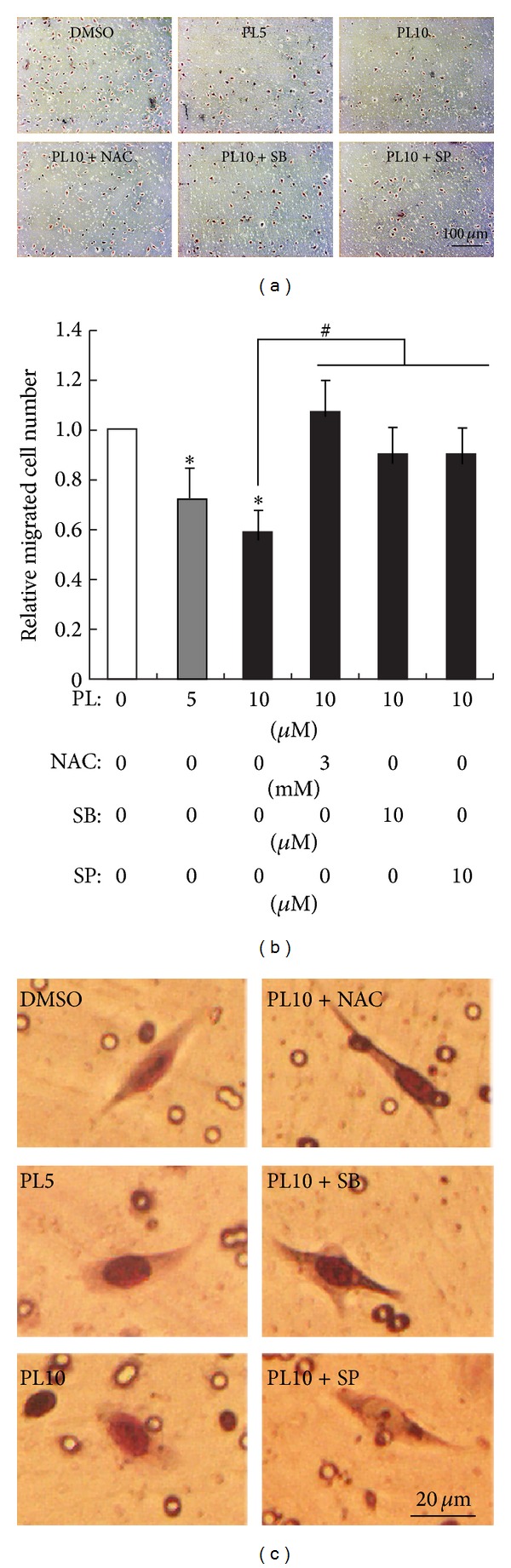
Effects of PL on LN229 cell migration in transwell cell migration assay. (a) Representative micrographs show the effects of PL on LN229 cell migration and the cell morphology of migrated LN229 cells. LN229 cells were seeded into the upper chamber of transwell apparatus in DMEM containing PL (0, 5, or 10 *μ*M) together with or without NAC (3 mM), SB203580 (10 *μ*M), or SP600125 (10 *μ*M). The medium in the lower chamber was replaced with DMEM containing 10% FBS in order to induce cell migration 6 h after PL incubation. Migrated cells in the lower surface of the filter were stained and microphotographed 24 h after cell migration induction by serum. (b) Statistical analysis of migrated cell numbers in different groups. *P < 0.05 compared to group of 0 *μ*M of PL (DMSO); ^#^ *P* < 0.05 compared to group of 10 *μ*M of PL treatment alone. (c) Morphology of migrated LN229 cells at higher magnification.

Further, we verified the effects of PL, NAC, p38, and JNK inhibitors on LN229 cell migration in another cell migration model, that is, the transwell migration assay. The results demonstrated that the numbers of LN229 cells migrating through the transwell were significantly reduced 24 h after PL treatment (5 and 10 *μ*M) (Figures 5(a) and 5(b)). Pretreatment of NAC, SB203580, or SP600125 significantly increased the migrated LN229 cells in the presence of PL (10 *μ*M) (Figures 5(a) and 5(b)). Concomitantly, the morphology of LN229 cells was changed from spindle shape with long processes to elliptical shape without evident process (enlarged micrographs in Figure 5(c)). Pretreatment of NAC, SB203580, or SP600125 resumed the spindle shape of migrated LN229 cells in the presence of PL (Figure 5(c)).

### 3.5. PL Inactivates NF*κ*B via ROS, p38, and JNK-Mediated Signaling

**Figure 6:**
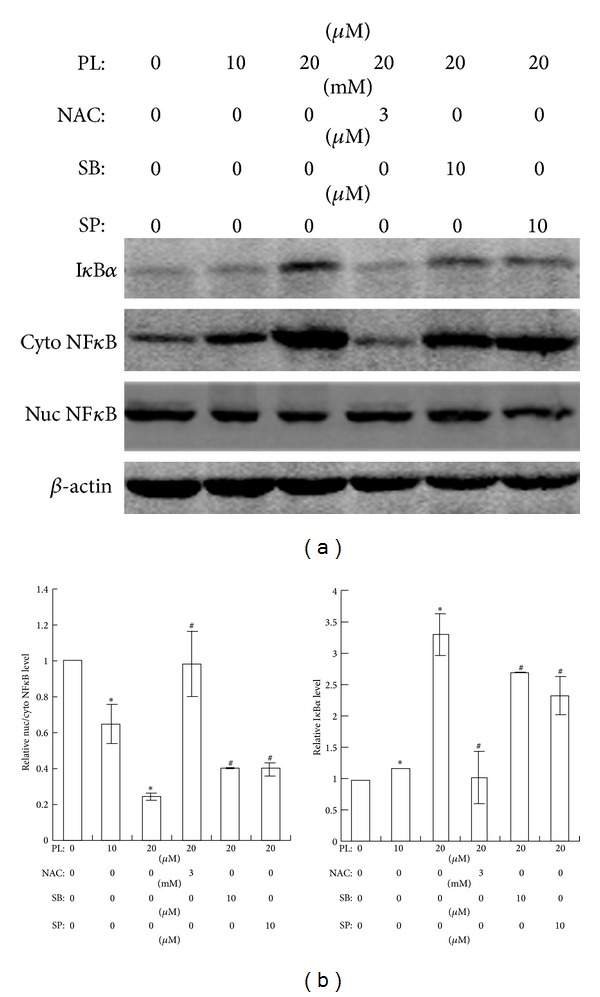
Effects of PL on NF*κ*B nuclear translocation and I*κ*B*α* expression in LN229 cells. (a) Representative western blots of NF*κ*B and I*κ*B*α* at 3 h after scratching in LN229 cells. Cells were pretreated with PL for 6 h; NAC, SB203580, or SP600125 was administrated 2 h before PL treatment. Cytosolic (cyto) and nuclear (nuc) protein were isolated and subjected to western blot analysis. *β*-actin was set as loading control. (b) Statistical analysis of nuc/cyto NF*κ*B of western blot results. Statistical analysis of I*κ*B*α* expression of western blot results. *P < 0.05 compared to group of 0 *μ*M of PL; ^#^ *P* < 0.05 compared to group of 20 *μ*M of PL treatment alone.

Since NF*κ*B is heavily involved in cancer metastasis and is considered to be a downstream target of ROS, p38, and JNK, we analyzed the nuclear translocation of NF*κ*B and the expression of I*κ*B*α*, which binds to NF*κ*B and retains it in the cytoplasm. Results of western blot clearly showed that I*κ*B*α* and cytoplasmic NF*κ*B (cyto NF*κ*B) were evidently increased in LN229 cells at 3 h after scratching upon 10 or 20 *μ*M PL while nuclear NF*κ*B (nuc NF*κ*B) was decreased as compared to vehicle control (0 *μ*M PL) (Figure 6(a)). Administration of NAC completely reversed the effects of PL on the I*κ*B*α* and cyto/nuc NF*κ*B expression while inhibitors of p38 (SB203580) or JNK (SP600125) partially attenuated PL's effects (Figure 6(a)). Statistical analysis demonstrated that relative nuc/cyto NF*κ*B ratio (representing NF*κ*B activity) was significantly reduced by PL while NAC, SB203580, or SP600125 could reverse PL's effect on NF*κ*B nuclear translocation (Figure 6(b)).

## 4. Discussion

In the present study, we have demonstrated that PL is effective in suppressing the migration of GBM cells. This anticancer effect of PL depends on enhanced ROS in LN229 cells. ROS-dependent p38/JNK activation and inhibition of NF*κ*B nuclear translocation contribute to PL's inhibitory effects on LN229 cell migration.

The migration of cancer cells is pivotal for cancer invasion and metastasis [30–32]. Cancer malignancy is proportional to cancer cell's migration ability. GBM is one of the most invasive cancers refractory for present therapy [21]. We found that PL at lower concentration (1 *μ*M) was effective in inhibiting LN229 and U87 cell migration while PL at higher concentration (10 *μ*M) did not interfere with the mobility of normal astrocytes (Figure 1(b)), suggesting that normal brain functions might not be disturbed during PL therapy. Since we measured cell migration within 6 h after the induction of migration at a lower PL dosage as compared to the half inhibitory concentration of PL in LN229 and U87 (10–20 *μ*mol/L) [22] and PL at 5 or 10 *μ*mol/L did not inhibit cell proliferation in scratch-wound model (Figure 2), therefore, the death and proliferation of LN229 was negligible in our assays. Moreover, we tested the ability of LN229 cell migration in two different systems. Thus, the effects of PL on cancer cell migration are reliable. Interestingly, migrated LN229 cells in transwell assay showed remarkable morphological changes (Figure 5(c)). It is well-known that cell migration requires cell deformation [31, 33]. The round shape of LN229 cells in the presence of PL might make them difficult to be transformed. It is reported that p38 activation could upregulate E-cadherin and downregulate N-cadherin and vimentin in malignant HaCaT cells [34], which are key molecules mediating cancer cell migration or invasion. It is likely that PL might alter the expression of cadherin or vimentin and thus induce the morphological changes in GBM cells. Further elucidating the mechanisms of cell deformation in the presence of PL is desirable.

ROS accumulation seems to be required for the anticancer effects of PL as verified in various studies since ROS is elevated and the use of antioxidant NAC completely abolishes all biological functions of PL in cancer cells [15, 17, 22, 28]. However, it is recently reported that NAC is not a specific antioxidant as it also suppresses the inhibitor of proteasome [35]. Thus, it is necessary to test further the effects of more antioxidants on PL's functions and elucidate the sources and mechanisms of ROS elevation in PL-treated cancer cells. In LN229 cells after scratch, cellular ROS was elevated while glutathione was reduced, suggesting that PL elevated ROS via impairing ROS clearance system. Until now, how PL increases cellular ROS remains unclear although it is suggested that the binding of PL to GSTP1 or CBR1 might contribute to PL-induced ROS [15].

The mechanisms underlying ROS's biological functions are extremely complicated [36]. Identifying key molecules downstream ROS-mediated signaling pathways in cancer cells is required for improving the therapeutic effects of drugs or developing novel anticancer reagents [37, 38]. In LN229 cells after the scratch injury, p38 and JNK were activated notably by PL. Since NAC completely abolished p38 and JNK activation, p38 and JNK were downstream targets of ROS elevation upon PL treatment [22]. Pharmacological assays demonstrated that the inhibition of p38 or JNK by specific inhibitors significantly reduced PL's effect on LN229 cell migration. Therefore, both p38 and JNK signaling pathways contributed to ROS-induced inhibition of LN229 cell migration in the presence of PL. Since p38 and JNK exert suppressive [39, 40] or promoting [41, 42] effects on cancer metastasis in different cellular contexts, further dissecting p38 and JNK signaling pathways in PL-treated GBM cells might be helpful for understanding the mechanisms of the anticancer effects of PL.

PL exerted prominent effects on reducing nuclear translocation of NF*κ*B and NAC completely reversed the inhibitory effect of PL on NF*κ*B activation. Considering the essential role of NF-*κ*B in cell migration via activating snail and repressing E-cadherin [43, 44], it is conceivable that PL might exert its effects on cell migration via ROS-NF*κ*B pathway. Since p38 and JNK activation depended on ROS accumulation and inhibitors of p38 or JNK could partially reverse the effects of PL on NF*κ*B activity, we speculated that p38 and JNK were intermediate players between ROS and NF*κ*B.

## 5. Conclusion

In summary, the natural compound piperlongumine can effectively and selectively suppress cancer cell migration. This action of PL depends on PL-induced ROS-p38/JNK-NF*κ*B signaling pathway. Our data suggest that PL is a potential therapeutic for highly invasive cancers such as GBM.
